# RELIGIOUS DIFFERENCE, MARITAL SATISFACTION, AND PSYCHOLOGICAL WELL-BEING OF CHINESE MIDDLE-AGED AND OLDER ADULTS

**DOI:** 10.1093/geroni/igz038.2301

**Published:** 2019-11-08

**Authors:** Jia Li, Qi Wang

**Affiliations:** 1 The Hong Kong Polytechnic University, Hong Kong, Hong Kong; 2 University of Hong Kong, Hong Kong, Hong Kong

## Abstract

Objectives: Religion plays an important role in people’s individual and interpersonal life. Spousal religious difference is a potential risk factor of marital satisfaction, which will further influence people’s psychological well-being. This study aims to explore the associations between spousal religious differences, marital satisfaction, and psychological well-being of Chinese middle-aged and older adults. We also investigated the gender differences in the captioned associations. Method: We adopted a sample of 1285 adults aged 45 and above from the China Health and Retirement Longitudinal Study (CHARLS). We conducted descriptive statistics, multiple regression models and a path analysis based on a general structural equation model (GSEM). Results: Spousal religious difference was only associated with wives’ marital satisfaction. Marital satisfaction was associated with depression and life satisfaction for both genders, and wives’ marital satisfaction had a stronger association with life satisfaction than husbands’. Wives’ marital satisfaction mediated the relationship between spousal religious difference and their psychological well-being, including depression and life satisfaction. Discussion: This study calls for more further research on the individual and interpersonal outcomes of religiosity in middle-aged and older adults. Gender differences should be paid attention to in academic research, service provision and clinical settings.

